# Clinical Course of Isolated Recurrent, Persistent Complex Perianal Fistulas Without Luminal Crohn’s Disease: A Multicenter Case Series of 24 Patients

**DOI:** 10.1093/crocol/otae065

**Published:** 2024-11-27

**Authors:** Hannah W Fiske, Chung Sang Tse, Badr Al-Bawardy, Pooja Magavi, Gauree Gupta Konijeti, Eric Mao, Sean Fine, Alyssa Parian, Mark Lazarev, Samir A Shah

**Affiliations:** Department of Internal Medicine, The Warren Alpert Medical School of Brown University, Providence, RI, USA; Division of Gastroenterology, Perelman School of Medicine at the University of Pennsylvania, Philadelphia, PA, USA; Department of Internal Medicine, Division of Gastroenterology and Hepatology, King Faisal Specialist Hospital and Research Center, Riyadh, Saudi Arabia; College of Medicine, Alfaisal University, Riyadh, Saudi Arabia; Division of Gastroenterology, Yale School of Medicine, New Haven, CT, USA; Division of Gastroenterology & Hepatology, Scripps Clinic, La Jolla, CA, USA; Division of Gastroenterology & Hepatology, Scripps Clinic, La Jolla, CA, USA; Division of Gastroenterology and Hepatology, University of California Davis School of Medicine, Sacramento, CA, USA; Division of Gastroenterology, The Warren Alpert Medical School of Brown University, Providence, RI, USA; Division of Gastroenterology & Hepatology, Johns Hopkins Hospital, Baltimore, MD, USA; Division of Gastroenterology & Hepatology, Johns Hopkins Hospital, Baltimore, MD, USA; Gastroenterology Associates Inc. (Powered by GI Alliance), The Warren Alpert Medical School of Brown University, Providence, RI, USA

**Keywords:** perianal fistula, isolated perianal Crohn’s disease, inflammatory bowel disease

## Abstract

**Background:**

Isolated complex perianal fistulas, without luminal evidence of inflammatory bowel disease in the gastrointestinal tract, pose diagnostic and treatment dilemmas for gastroenterologists and colorectal surgeons. For patients who develop recurrent complex fistulas, a presumptive diagnosis of Crohn’s disease may be made. It is unclear whether these cases of isolated perianal disease in the absence of luminal inflammation truly represent isolated severe cryptoglandular fistulas or rather an early presentation of Crohn’s disease. We aimed to investigate the clinical course and outcomes of patients with isolated complex perianal fistulas.

**Methods:**

In this retrospective multicenter case series across 6 institutions in the United States, we report the clinical course of patients with isolated recurrent complex perianal fistulas, including their diagnostic evaluation, medical and surgical therapies, and clinical outcomes.

**Results:**

All patients (*n* = 24) required incision and drainage of perirectal abscesses. The majority received setons (*n* = 19, 79%), more intensive surgical interventions (*n* = 15, 62.5%, including fistulotomy/sphincterotomy, advancement flap, and ligation of the intersphincteric fistula tract), antibiotics (*n* = 17, 71%), and biologic therapy (*n* = 16, 67%). Nine patients (37.5%) underwent a combined medical-surgical approach with biologics and intensive surgical intervention. Despite surgical and/or medical management, active symptomatic complex perianal fistulas persisted in 58% (*n* = 14) of patients at follow-up (median 5.5 years, interquartile range 2.5-10 years); symptom remission was achieved in 21% (*n* = 5), and fistula closure in 21% (*n* = 5).

**Conclusions:**

These cases highlight a multidisciplinary and multimodal approach when treating isolated complex perianal fistulas and their propensity to persist despite the incorporation of advanced therapies.

## Graphical Abstract

**Graphical Abstract:**
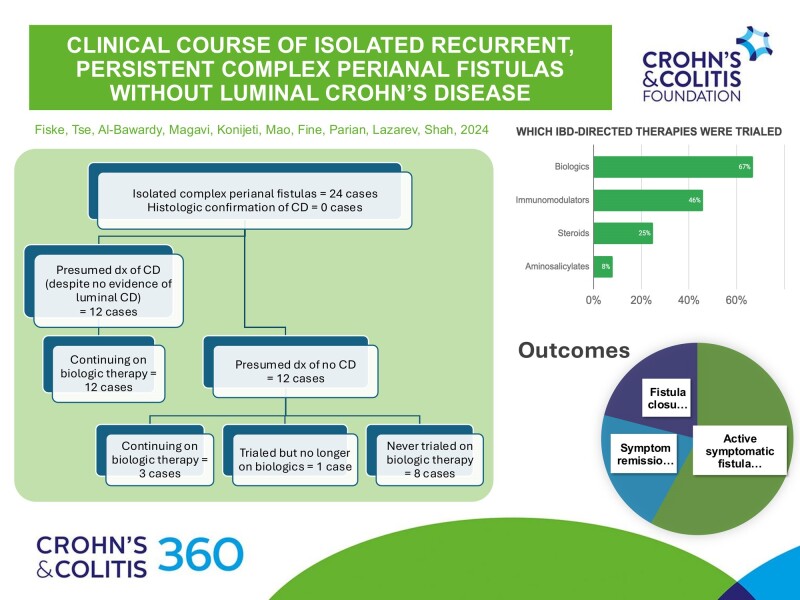

## Introduction

Complex perianal fistulas are abnormal connections between the epithelial surfaces of the anal canal and the perianal area above the dentate line (intersphincteric, transsphincteric, extrasphincteric, or suprasphincteric) and may be associated with multiple external openings, may involve the rectum or vagina, and may be present with perianal abscess or anal stenosis.^1^ Based on data from population-based studies, between 20% and 25% of patients with Crohn’s disease (CD) typically develop perianal fistulas, though isolated perianal CD is rare.^2,3^ To add to the diagnostic dilemma, an initial presentation of isolated complex perianal fistula may precede the development of luminal CD. In a single-center study of 13 patients with isolated perianal Crohn’s disease, 4 (31%) had persistent fistulas after a median 10-year follow-up between 1980 and 2000, prior to the biologic era.^4^ Empirical evidence is lacking on the clinical course of isolated complex perianal fistulas in the 21st century, whereupon the introduction and widespread use of biologic therapies and advancement of surgical techniques changed the landscape of CD care.^5,6^ In this study, we aimed to investigate the clinical course and outcomes of patients with isolated complex perianal fistulas.

## Materials and Methods

We herein describe a case series of the clinical course of 24 adults with isolated complex perianal fistula without luminal CD, treated with a combination of medical and surgical therapies from 2004 to 2024. These cases are uncommon and can be difficult to treat, so stand out to their gastroenterologists. In this case series, patients were identified by their providers by a variety of methods: ICD-10 code search (*n* = 9, 37.5%), physician recall (*n* = 9, 37.5%), or opportunity/convenience sampling (*n* = 6, 25%; the provider took note whenever patients with isolated perianal fistulas presented to clinic or endoscopy; they recorded the patients’ MRNs in a private list within the EMR as they were identified). Patients were subsequently evaluated via chart review and were determined to have an absence of luminal inflammation based on a combination of ileocolonoscopy, cross-sectional imaging, and capsule endoscopy.

### Ethical Considerations

This study is original and has not been published previously. All authors significantly contributed to the design of the study, data analysis, and drafting and approved the final version of the manuscript. All authors agree to be accountable for all aspects of this study.

This study was reviewed and approved by the Institutional Review Board (protocol #IRB00210084).

## Results

Twenty-four adults (9 male and 15 female) with a median age of 33 years (interquartile range [IQR] 27.5-42 years) and no prior gastrointestinal (GI) diseases presented between 2004 and 2024 to 6 academic medical centers in the United States. On exam, each patient was found to have an isolated complex perianal fistula in the absence of any luminal findings of CD (Table 1). Presenting symptoms were perianal pain (*n* = 22, 92%), abdominal pain (*n* = 6, 25%), diarrhea (*n* = 6, 25%), and arthralgia (*n* = 4, 17%). None of the patients had evidence of inflammation on colonoscopy, colonic biopsies, or imaging via magnetic resonance imaging (MRI) or computed tomography (CT). Eight patients (33%) underwent esophagogastroduodenoscopy (EGD), ruling out upper GI Crohn’s. Eleven patients (46%) underwent discrete small bowel imaging with either computed tomography enterography (CTE), magnetic resonance enterography (MRE), or video capsule endoscopy (VCE), with no evidence of luminal GI inflammation.

**Table 1.** Summary table.

PT #	Initial presentation	Diagnostic evaluations	Subsequent diagnosis	Medical therapies attempted	Surgical procedures	Ultimate diagnosis and plan
Cases with a current presumptive diagnosis of CD, despite lack of evidence of luminal CD:						
*Continuing on biologic therapy*						
(1) 34M	Abdominal pain, diagnosed with sigmoid diverticulitis	Colonoscopy + bx CT MRI MRE EUA	Refractory perianal fistula with abscess	**Biologics, aminosalicylates, and immunomodulators:** Infliximab x16mo (continuing) Ustekinumab x10mo Tacrolimus Azathioprine **Non-CD medical therapies:** Metronidazole Ciprofloxacin Amoxicillin-Clavulanic acid	I&D Seton	**Outcome:** Active perianal fistula **Diagnosis:** Isolated perianal disease in the setting of presumed CD **Timeline:** 13 years after initial presentation **Treatment Plan:** Chronic tx with chronic, cyclical abx (Ciprofloxacin or Amoxicillin-Clavulanic acid) CD-directed therapy (Azathioprine + Infliximab; will consider Upadacitinib once approved by the FDA) Planning for surgical f/u
(2) 24F	Perianal pain, diagnosed as perianal abscess with fistula; also presented with rheumatologic symptoms	Colonoscopy + bx MRI EUA	Refractory perianal abscess; inflammatory arthropathy	**Biologics, aminosalicylates, and immunomodulators:** Adalimumab x46mo Infliximab x12mo (continuing) Azathioprine **Non-CD medical therapies:** Ciprofloxacin Metronidazole	I&D Seton	**Outcome:** Active perianal fistula **Diagnosis:** Presumed CD with perianal disease and inflammatory arthropathy **Timeline:** 9 years after initial presentation **Treatment Plan:** CD-directed therapy (Infliximab + Azathioprine) Planning for surgical f/u
(3) 50F	Diarrhea and rectal ulcers, presumed to have CD; also presented with rheumatologic symptoms	Colonoscopy + bx (including ileal and rectal bx) CT MRI EUA	Refractory perianal fistula with abscess	**Biologics, aminosalicylates, and immunomodulators:** Infliximab x39mo (continuing) **Non-CD medical therapies:** Colchicine Prednisone	I&D Seton Perineal debridement Fistulotomy	**Outcome:** Active perianal fistula **Diagnosis:** Presumed CD with perianal disease **Timeline:** 5 years after initial presentation **Treatment Plan:** CD-directed therapy (Infliximab)
(4) 21F	Ischiorectal abscess and posterior anal fissure, diagnosed with isolated perianal CD	Colonoscopy + bx (including ileal and rectal bx) MRI EUA Fecal calprotectin (within normal limits)	Isolated perianal CD	**Biologics, aminosalicylates, and immunomodulators:** Infliximab x16mo (continuing) Mercaptopurine **Non-CD medical therapies:** Ciprofloxacin Metronidazole	I&D Seton Transanal advancement flap Penrose drain placement Foley catheter placed into perianal abscess cavity Diverting colostomy	**Outcome:** Active perianal fistula **Diagnosis:** Presumed isolated perianal CD and lymphocytic colitis **Timeline:** 6 years after initial presentation **Treatment Plan:** CD-directed therapy (Infliximab + Mercaptopurine)
(5) 34F	Abdominal pain, constipation, and hematochezia, given presumptive diagnosis of unspecified IBD	Colonoscopy + bx (including ileal and rectal bx) US MRI MRE EUA Fecal calprotectin (mild elevation)	Refractory perianal fistula with abscess	**Biologics, aminosalicylates, and immunomodulators:** Ustekinumab x9mo (continuing) Mesalamine **Non-CD medical therapies:** Trimethoprim-Sulfamethoxazole	I&D Seton	**Outcome:** Active perianal fistula **Diagnosis:** Presumed unspecified IBD with refractory perianal disease **Timeline:** 4.5 years after initial presentation **Treatment Plan:** CD-directed therapy (Ustekinumab)
(6) 16F	Isolated perianal fistula with abscess, presumed to have isolated perianal CD; family history of Crohn’s disease	Colonoscopy + bx (including ileal and rectal bx) MRI EUA Fecal calprotectin (elevated)	Isolated perianal CD	**Biologics, aminosalicylates, and immunomodulators:** Infliximab x64mo (continuing) **Non-CD medical therapies:** Ciprofloxacin Metronidazole	I&D Seton	**Outcome:** Active perianal fistula **Diagnosis:** Presumed isolated perianal CD **Timeline:** 5.5 years after initial presentation **Treatment Plan:** CD-directed therapy (Infliximab)
(7) 36M	Chronic diarrhea, abdominal pain, and isolated perianal fistula with abscess presumed to have isolated perianal CD; also presented with spondyloarthropathy.	EGD Colonoscopy + bx MRE EUA	Isolated perianal CD	**Biologics, aminosalicylates, and immunomodulators:** Infliximab x16mo Ustekinumab x32mo (continuing) Azathioprine **Non-CD medical therapies:** Ciprofloxacin	I&D Seton Diverting loop ileostomy LIFT Stem cell injections	**Outcome:** Active perianal fistula **Diagnosis:** Presumed isolated perianal CD **Timeline:** 18 years after initial presentation **Treatment Plan:** CD-directed therapy (Ustekinumab with stem cell injections)
(8) 43M	Perianal fullness and pain, with episodes of drainage, diagnosed with recurrent perianal fistula	Colonoscopy + bx MRI EUA	Isolated perianal CD	**Biologics, aminosalicylates, and immunomodulators:** Infliximab x44mo (continuing) **Non-CD medical therapies:** Ciprofloxacin	I&D Seton	**Outcome:** Active perianal fistula **Diagnosis:** Presumed isolated perianal CD **Timeline:** 5 years after initial presentation **Treatment Plan:** CD-directed therapy (Infliximab) Planning for surgical f/u
(9) 30F	Pilonidal cyst with anal fistula	EGD Colonoscopy + bx (including ileal and rectal bx) MRI Fecal calprotectin (mild elevation)	Complex, recurrent perianal fistula	**Biologics, aminosalicylates, and immunomodulators:** Infliximab x17mo Mercaptopurine Adalimumab x50mo (continuing) **Non-CD medical therapies:** Ciprofloxacin Metronidazole Amoxicillin-Clavulanic acid Budesonide	I&D Seton Cystectomy Fistulotomy	**Outcome:** Fistula symptom remission **Diagnosis:** Presumed chronic fistulizing perianal CD; in steroid-free clinical and biochemical remission **Timeline:** 9 years after initial presentation **Treatment Plan:** CD-directed therapy (Adalimumab)
(10) 27M	Perianal abscess in the setting of long history of anal fissures; family history of Crohn’s disease	Colonoscopy + bx (including ileal and rectal bx) MRI MRE	Isolated perianal CD	**Biologics, aminosalicylates, and immunomodulators:** Infliximab x24mo Mercaptopurine x6mo	I&D Seton Sphincterotomy	**Outcome:** Fistula closure (clinical closure) **Diagnosis:** Presumed isolated perianal CD; no recurrence of fistula or fissure **Timeline:** 2.5 years after initial presentation **Treatment Plan:** CD-directed therapy (Infliximab)
(11) 41F	Perianal fistula in the setting of long history of abdominal pain and alternating diarrhea and constipation; family history of Crohn’s disease	EGD Capsule Flexible sigmoidoscopy Colonoscopy + bx (including rectal bx) CTE EUA	Unclear dx	**Biologics, aminosalicylates, and immunomodulators:** Adalimumab x1mo (continuing) **Non-CD medical therapies:** Undefined abx	I&D Seton	**Outcome:** Active perianal fistula **Diagnosis:** Presumed isolated perianal CD; continues to develop recurrent fistulas **Timeline:** 1 year after initial presentation **Treatment Plan:** CD-directed therapy (adalimumab) Undefined abx
(12) 28F	Perianal fistula with abscess developed after birth of first child, had previous history of recurrent abscesses; diagnosed with presumed isolated perianal CD	Colonoscopy + bx (including ileal and rectal bx) MRI MRE CT EUA Fecal calprotectin (within normal limits)	Still considering as isolated perianal CD despite lack of luminal disease and normal fecal calprotectin	**Biologics, aminosalicylates, and immunomodulators:** Adalimumab x7mo Infliximab x41mo (continuing) Azathioprine **Non-CD medical therapies:** Amoxicillin-clavulanic acid	I&D Seton Mallinckrodt drain Fistulotomy x2	**Outcome:** Fistula symptom remission **Diagnosis:** Presumed isolated perianal CD; MRI shows healing of fistula **Timeline:** 7 years after initial presentation **Treatment Plan:** CD-directed therapy (Infliximab), planning to discontinue with close monitoring in the near future given MRI showing healing
Cases with no current presumptive diagnosis of CD						
*Continuing on biologic therapy*						
(13) 69M	Recurrent diverticulitis with perianal abscess and fistula	EGD Colonoscopy + bx (including ileal and rectal bx) CT CTE MRI MRE Fecal calprotectin (elevated)	Ileitis with recurrent perianal disease	**Biologics, aminosalicylates, and immunomodulators:** Adalimumab x11mo (continuing)	I&D Seton LIFT	**Outcome:** Active perianal fistula **Diagnosis:** Ileitis with recurrent perianal disease, no luminal CD **Timeline:** 13 years after initial presentation **Treatment Plan:** CD-directed therapy (Adalimumab) MRE pending
(14) 30F	Perianal fistula; family history of Crohn’s disease	EGD Colonoscopy + bx (including ileal and rectal bx) MRI Fecal calprotectin (within normal limits)	Ileitis with recurrent perianal abscess	**Biologics, aminosalicylates, and immunomodulators:** Adalimumab x52mo (continuing)	I&D FMT	**Outcome:** Fistula symptom remission **Diagnosis:** Ileitis with recurrent perianal abscess, no luminal CD, complicated by recurrent Clostridioides difficile; clinically in symptom remission **Timeline:** 8 years after initial presentation **Treatment Plan:** CD-directed therapy (Adalimumab) FMT
(15) 18F	Perianal pain found to have perianal fistula w/ abscess, no other GI symptoms; some intermittent rheumatologic sx	EGD Colonoscopy + bx (including ileal and rectal bx) Flexible sigmoidoscopy CT MRI MRE EUA Fecal calprotectin (within normal limits)	Unclear dx	**Biologics, aminosalicylates, and immunomodulators:** Infliximab x53mo **Non-CD medical therapies:** Amoxicillin Hyperbaric oxygen	I&D Seton Mushroom drain Fistulotomy	**Outcome:** Fistula symptom remission **Diagnosis:** Isolated perianal disease **Timeline:** 5.5 years after initial presentation **Treatment Plan:** CD-directed therapy (Infliximab)
*Trialed but no longer on biologic therapy*						
(16) 39F	Painful perianal mass, presumed to be an abscess or hemorrhoids	Flexible sigmoidoscopy Colonoscopy + bx (including rectal bx) MRI EUA	Refractory perirectal and rectovaginal fistula with abscess (diagnosed following childbirth via cesarean section, not vaginal delivery)	**Biologics, aminosalicylates, and immunomodulators:** Adalimumab x9mo Methotrexate **Non-CD medical therapies:** Ciprofloxacin Metronidazole Amoxicillin-Clavulanic acid Prednisone	I&D Seton Fistulectomy + sphincterotomy Diverting sigmoid colostomy Fistulotomy	**Outcome:** Active perianal fistula **Diagnosis:** Do not suspect CD; nonhealing cryptoglandular anal fistula c/b surgery **Timeline:** 3 years after initial presentation **Treatment Plan:** Discontinued CD therapy given lack of clinical improvement; off all medical tx Planning for surgical f/u with consideration of transperitoneal repair vs clinical trial of stem cell injection
*Never trialed on biologic therapy*						
(17) 43M	Painful perianal mass, presumed to be a rectal abscess	Colonoscopy + bx MRI EUA	Refractory perianal abscess	**Non-CD medical therapies:** Amoxicillin-Clavulanic acid	I&D	**Outcome:** Fistula symptom remission **Diagnosis:** Isolated cryptoglandular abscess with proctitis and internal hemorrhoids **Timeline:** 2.5 years after initial presentation **Treatment Plan:** No need for chronic antibiotics; off all medical tx Sitz baths and Preparation H as needed If symptoms worsen will plan for surgical f/u
(18) 28F	Perianal abscess and fistula in ano in the setting of lifelong diarrheal illness and known lymphocytic colitis, concern for CD; family history of ulcerative colitis	Colonoscopy + bx (including ileal and rectal bx) CT MRI EUA Fecal calprotectin (mild elevation)	Microscopic colitis with refractory perianal fistula with abscess	**Biologics, aminosalicylates, and immunomodulators:** Balsalazide Mesalamine **Non-CD medical therapies:** Amitriptyline Bismuth Subsalicylate Budesonide CBD oil	I&D Seton Collagen plug placement Fibrin glue Fistulotomy Cystoscopy + laser lithotripsy Stem cell injection	**Outcome:** Active perianal fistula **Diagnosis:** Microscopic colitis **Timeline:** 13 years after initial presentation **Treatment Plan:** Continuing mesenchymal stem cell injections as part of clinical trial
(19) 32M	Perianal fistula with drainage; family history of ulcerative colitis	EGD Colonoscopy + bx (including ileal and rectal bx) VCE MRE EUA Fecal lactoferrin (mild elevation)	Refractory perianal fistula with abscess	**Biologics, aminosalicylates, and immunomodulators:** Methotrexate **Non-CD medical therapies:** Budesonide	I&D Seton Fistulotomy Advancement flap procedure LIFT	**Outcome:** Active perianal fistula **Diagnosis:** Refractory perianal dz **Timeline:** 12 years after initial presentation **Treatment Plan:** Budesonide taper with plan to start CD-directed therapy (anti-TNF combination therapy) Patient was unfortunately lost to follow-up
(20) 35F	IBS, rectal pain and perianal drainage	EGD Flexible sigmoidoscopy Colonoscopy + bx (including ileal and rectal bx) VCE MRI EUA Fecal calprotectin (within normal limits)	Complex perianal fistula	**Biologics, aminosalicylates, and immunomodulators:** Methotrexate **Non-CD medical therapies:** Ciprofloxacin Metronidazole Budesonide	I&D Seton Endorectal advancement flap Fistulotomy	**Outcome:** Fistula closure (clinical closure) **Diagnosis:** Isolated perianal disease; status post successful surgery with closure of fistula **Timeline:** 11 years after initial presentation **Treatment Plan:** Not on treatment
(21) 59F	Pilonidal cyst, found to have 2 draining perianal fistulas; family history of Crohn’s disease	Capsule Flexible sigmoidoscopy Colonoscopy + bx (including rectal bx) MRI EUA	Isolated perianal CD	**Biologics, aminosalicylates, and immunomodulators:** Mercaptopurine x6-9mo	I&D Fistulotomy	**Outcome:** Fistula closure (clinical + radiologic closure) **Diagnosis:** Cryptoglandular fistula, no luminal CD; no further drainage, fistulas closed **Timeline:** 1.5 years after initial presentation **Treatment Plan:** Not on treatment
(22) 44M	Recurrent rectal abscess	Colonoscopy + bx (including ileal and rectal bx) CT MRI MRE EUA Fecal calprotectin (elevated)	Unclear dx	**Non-CD medical therapies:** Ciprofloxacin Metronidazole	I&D Seton Endoanal advancement flap	**Outcome:** Fistula closure (clinical and radiologic closure) **Diagnosis:** Cryptoglandular fistula, no luminal CD; abscess resolved w/o fistula **Timeline:** 1 year after initial presentation **Treatment Plan:** Not on treatment
(23) 22M	Perirectal abscess	Colonoscopy + bx MRI EUA	Unclear dx	**Non-CD medical therapies:** Undefined abx	I&D	**Outcome:** Fistula closure (clinical closure) **Diagnosis:** Cryptoglandular fistula, no luminal CD; abscess resolved w/o fistula **Timeline:** 1 year after initial presentation **Treatment Plan:** Not on treatment
(24) 28F	Perianal abscess in the setting of abdominal pain and intermittent diarrhea	Colonoscopy + bx (including ileal and rectal bx) MRI EUA	Unclear dx	**Non-CD medical therapies:** Undefined abx	I&D Fistulotomy + marsupialization	**Outcome:** Active perianal fistula **Diagnosis:** Cryptoglandular fistula, no luminal CD; persistent fistula **Timeline:** 1 year after initial presentation **Treatment Plan:** Undefined abx

Abbreviations: Bx, biopsies; C/B, complicated by; CD, Crohn’s disease; EUA, exam under anesthesia; F, female; FMT, fecal microbiota transplantation; F/U, follow-up; I&D, incision and drainage; IBD, inflammatory bowel disease; IBS, irritable bowel syndrome; LIFT, ligation of the intersphincteric fistula tract; M, male; MRE, magnetic resonance elastography; MRI, magnetic resonance imaging; TNF, tumor necrosis factor; VCE, video capsule endoscopy.

**Figure 1.**
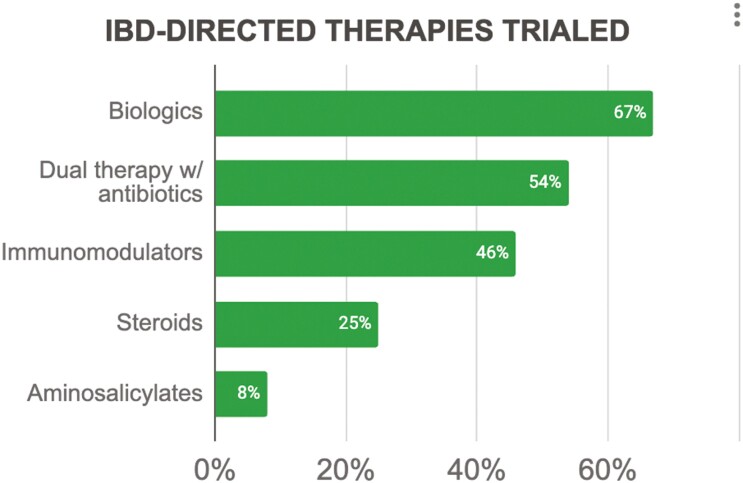
The IBD-directed therapies which were trialed.

All patients received medical therapy, with just over half receiving a combination of antibiotics and CD-directed therapies (*n* = 13, 54%), 17% receiving antibiotics alone (*n* = 4), and 29% receiving CD-directed therapies alone (*n* = 7). The most common antibiotics used were ciprofloxacin (*n* = 10, 42%), metronidazole (*n* = 8, 33%), and amoxicillin/amoxicillin-clavulanic acid (*n* = 6, 25%) (Table 1). Crohn’s disease-directed therapies (Figure 1) included biologics (*n* = 16, 67%; dual therapy with antibiotics: *n* = 13, 54%), immunomodulators (*n* = 11, 46%), and aminosalicylates (*n* = 2, 8%); steroids were used in 25% (*n* = 6) (Table 1). Biologic therapy was continued for a mean of 17 months (range 1-64 months), and included infliximab (*n* = 11, 46%; median 24 months, IQR 16-44 months), adalimumab (*n* = 7, 29%; median 11 months, IQR 7-50 months), and ustekinumab (*n* = 3, 12.5%; median 10 months, IQR 9.5-21 months). Combination therapy with biologics and immunomodulators was used for 33% (*n* = 8) of the patients.

All patients had exam under anesthesia (EUA) with incision and drainage (I&D) of perirectal abscesses. Setons were placed in 79% (*n* = 19) of patients. More intensive surgical interventions were pursued in 62.5% (*n* = 15) of patients, with nearly half of all patients (*n* = 10, 42%) undergoing fistulotomy, while < 20% underwent advancement flap (*n* = 4, 17%) or ligation of the intersphincteric fistula tract (LIFT) (n = 3, 12.5%) (Table 1). Two patients (8%) were trialed on stem cell therapy via injection. Overall, 9 patients (37.5%) had a combined medical-surgical approach, undergoing treatment with both biologics and more intensive surgical interventions.

We examined the proportion of patients with active perianal fistula (persistent perianal drainage and/or recurrent abscess), fistula symptom remission (cessation of drainage and no abscess) and fistula closure (clinical and/or radiological closure of the fistula tract) on follow-up. Despite surgical and medical management, active perianal fistula was noted in 58% (*n* = 14) of patients after a median of 5.5 years (range 1-18 years, IQR 2.5-10 years). Fistula symptom remission was achieved in 21% (*n* = 5), and full fistula closure in 21% (*n* = 5; all 5 with fistula closure on clinical exam, and 2 of the 5 with confirmed radiologic closure). Of the 10 patients who achieved symptom remission or fistula closure (Table 1), 70% (*n* = 7) had undergone more definitive surgical closure with fistulotomy or sphincterotomy (*n* = 6) or advancement flap (*n* = 2). Half (*n* = 5, 50%) had been treated with biologic therapy throughout the duration of the follow-up period; 40% (*n* = 4) had combination therapy with both biologics and more intensive surgical intervention.

Among the 9 patients who had been treated with a combined approach of biologics and intensive surgical interventions, 44% (*n* = 4) achieved fistula symptom remission (*n* = 3) or fistula closure (*n* = 1). Of the 15 patients who underwent more intensive surgical interventions, 47% (*n* = 7) achieved fistula symptom remission (*n* = 3) or fistula closure (*n* = 4). For the 16 patients trialed on biologics, 15 (94%) continued biological treatment at the end of the follow-up period, though only 5 (31%) achieved fistula symptom remission (*n* = 4) or fistula closure (*n* = 1). Of those 5 patients, 3 had success with infliximab and 2 with adalimumab; all 5 are continuing with biologic monotherapy. Eight patients were never trialed on biologics; of these patients, 3 continue to have active perianal fistula, 4 had successful surgeries resulting in fistula closure, and 1 is in symptomatic remission.

While 50% (*n* = 12) of the patients currently carry a presumptive diagnosis of CD (ie, presumed isolated perianal CD with complex fistula; some labeled with CD for obtaining prior authorization to use biologics), none to date have histologic confirmation of CD. Of the other 12 patients without a presumed CD diagnosis, 2 (8% of the total) were found to have persistent nonspecific ileitis without clear histologic evidence of CD. Although neither of these 2 patients is currently suspected to have CD, both are being continued on biologic therapy. While ileitis is often caused by CD, there are numerous mimickers that ought to be considered in patients without histologic confirmation of CD. Ileitis may be secondary to infectious disease, vasculitis, infiltrative disorders, ischemia, malignancy, or medications, among others.

## Discussion

Isolated perianal disease describes complex perianal fistulas in the absence of the luminal inflammation characteristically found in CD. Etiologies of isolated complex perianal fistulas ruled out for the patients in this study, include hidradenitis suppurativa, tuberculosis, actinomycosis, lymphogranuloma venereum, HIV, diverticulitis, postoperative fistula, trauma associated with childbirth, pelvic malignancy or subsequent irradiation therapy, and cryptoglandular abscess.^7,8^ Cryptoglandular abscesses are formed when there is impaired drainage of anal glands, leading to infection and formation of perianal abscesses. If abscesses are incompletely drained, then fistulas form.^8^ These fistulas, occurring in the absence of an underlying disease process, are typically simple fistulas (singular, transient, and low-lying). Recurrent complex fistulas are often presumed to be perianal CD and therefore may end up being treated with biologic therapy.

A diagnostic dilemma remains as to whether isolated perianal disease represents severe cryptoglandular fistula or CD (either an isolated perianal phenotype or an early manifestation prior to luminal disease).^9^ More than a third of patients with small bowel CD develop perianal symptoms before overt luminal disease.^10–12^ In a population-based cohort study of 85 CD patients with perianal or rectovaginal fistulas, 40% of patients developed their first fistula before or at the time of initial diagnosis of CD.^13^

For patients newly presenting with fistulas, diagnostic evaluation encompasses radiographic studies and direct visualization techniques. These tools may be employed to further evaluate the fistula (eg, EUA, proctosigmoidoscopy, or endoscopic anorectal ultrasound [US]), examine for luminal inflammation (eg, VCE), or both (eg, CT scan or pelvic MRI with contrast). Video capsule endoscopy can be a useful tool to identify mild small bowel disease that was not picked up on CT or MR.^10^ In a case series of 25 patients with persistent perianal disease without evidence of inflammation on colonoscopy, VCE revealed 24% of patients had aphthous ulcerations, linear ulcers, and circumferential ulcers indicative of active CD.^10^ Exam under anesthesia is another invaluable tool for the physician, with a diagnostic accuracy of 90% for fistulizing perianal CD.^2^ Exam under anesthesia allows for simultaneous surgical treatments, such as abscess I&D, seton placement, fistulotomy, fistulectomy, and as a last resort, proctectomy or colectomy.

Perianal fistulas are treated medically and surgically. In a year-long study of 22 patients with isolated perianal disease and 44 with perianal CD, treatment with tumor necrosis factor (TNF)-antagonists resulted in fistula healing in 43% of patients with perianal CD and 19% of patients with isolated perianal disease.^9^ This corresponds to the 31% response rate to biologics in our present case series of patients with isolated perianal disease. The use of steroids in patients without a clear diagnosis of luminal CD is not recommended; in fact, corticosteroids have been shown to decrease fistula healing.^14^ While treatment options for perianal fistulas are varied, relapse is common; in CD patients, data has shown that only 1/3rd of patients achieve remission of their fistulas.^4,15^

## Conclusions

This case series underscores the difficulty of managing isolated perianal fistulas and highlights the importance of considering a multimodal approach to treatment. While anti-TNF agents did not consistently result in fistula symptom remission or fistula closure, the findings from this case series suggest that their incorporation into the patient’s treatment regimen may be critical in achieving eventual remission. There were several limitations to this study. The small sample size (*n* = 24) naturally limits the generalizability of our findings. Given the rarity of isolated perianal fistulas, each patient in this study was memorable and stood out easily to the referring gastroenterologist. However, 37.5% of the patients were identified from provider memory alone; the nature of this method of patient identification can result in a recall/selection bias. The absence of luminal inflammation was determined via ileocolonoscopy, cross-sectional imaging, and capsule endoscopy; as this was a retrospective case series, examinations were not uniform across patients within this study. We cannot exclude the possibility of future development of luminal CD in this case series, though on repeat evaluations (median of 5.5 years [IQR 2.5-10 years]) none of the patients had yet to develop luminal disease. Of note, however, 15 of the patients discussed have been maintained on biologic therapy, which could be a factor in their disease control and the continued absence of luminal findings. Additionally, not all patients were subject to thorough and consistent small bowel evaluation, so our follow-up time does not preclude the missed diagnosis of small bowel CD. Given the similarity in management, it is important to consider CD in the differential for isolated perianal disease patients even without luminal findings.

## Data Availability

The data underlying this article are available within the article itself. Any additional data will be shared on reasonable request to the corresponding author.
